# IL-5, IL-8 and tgf-beta expressions in chronic rhinosinusitis patients with nasal polyps and their correlation to tissue's cellularity and disease relapsing

**DOI:** 10.1186/2045-7022-3-S2-P14

**Published:** 2013-07-16

**Authors:** Wilma T Anselmo-Lima, Aline J Gallego, Fransérgio E Cavallari, Daniel S Küpper, Cristiane Milanezi, João S Silva, Siomara Bambirra Oliveira, Franscesca M Faria, Edwin Tamashiro, Fabiana CP Valera

**Affiliations:** 1 School of Medicine of Ribeirão Preto - University of São Paulo, Ribeirão Preto, Brazil; 2 School of Medicine of Ribeirão Preto - University of São Paulo, ENT Division, Ribeirão Preto, Brazil; 3 School of Medicine of Ribeirão Preto - University of São Paulo, Biochemistry Department, Ribeirão Preto, Brazil; 4 School of Medicine of Ribeirão Preto - University of São Paulo, Pathology Department, Ribeirão Preto, Brazil

## Introduction

Chronic Rhinosinusitis with Nasal Polyps (CRSwNP) presents a Th2 profile predominance, with a remarkable eosinophil infiltration and IL-5 secretion. The influence of molecular markers on both histology and recurrence is still misunderstood.

## Aims

To evaluate the gene expression of IL-5, IL-8 and TGF-beta in patients with nasal polyps and control patients, as well as its correlation to tissue cellularity and diseases relapsing.

## Methods

The mRNA expression of the IL-5, IL-8 and TGF-beta protein was analyzed using qRT-PCR in 32 nasal polyps and 7 control samples comparing it with medium turbinate control samples (obtained from patients undergoing aesthetic rhinoplasty). The numbers of eosinophils and neutrophils were counted in samples obtained during surgical procedure, and stained with hematoxylin eosin in an optical microscope. The patients were followed up to three years after surgery, and considered with relapsing disease if there was polyp in the medium meatus through nasal endoscopy.

## Results

IL-5 and IL-8 expressions were increased whereas TGF-beta was decreased in the nasal polyps when compared to the control mucosa from medium turbinates. There was no correlation between these molecules expression and tissue's cellularity, nor to relapsing of the disease.

## Conclusion

Brazilians' nasal polyps present an increased Th2 and a decreased Treg profile, as Europeans'. The expression of none of these molecules correlated to tissue cellularity, neither to recurrence of the disease. Future research is still needed to observe the real impact of these molecules in CRSwNP, especially when regarding its therapy.

